# The Role of Local Inflammation and Hypoxia in the Formation of Hypertrophic Scars—A New Model in the Duroc Pig

**DOI:** 10.3390/ijms24010316

**Published:** 2022-12-24

**Authors:** Sebastian P. Nischwitz, Julia Fink, Marlies Schellnegger, Hanna Luze, Vladimir Bubalo, Carolin Tetyczka, Eva Roblegg, Christian Holecek, Martin Zacharias, Lars-Peter Kamolz, Petra Kotzbeck

**Affiliations:** 1 Division of Plastic, Aesthetic and Reconstructive Surgery, Department of Surgery, Medical University of Graz, 8036 Graz, Austria; 2 COREMED—Cooperative Centre for Regenerative Medicine, JOANNEUM RESEARCH Forschungsgesellschaft mbH, 8010 Graz, Austria; 3 Biomedical Research Unit, Medical University of Graz, 8036 Graz, Austria; 4 Department of Pharmaceutical Technology and Biopharmacy, Institute of Pharmaceutical Sciences, University of Graz, 8010 Graz, Austria; 5 HEALTH—Institute for Biomedicine and Health Sciences, JOANNEUM RESEARCH Forschungsgesellschaft mbH, 8010 Graz, Austria; 6 Diagnostic and Research Institute of Pathology, Medical University of Graz, 8036 Graz, Austria; 7 Research Unit for Tissue Regeneration, Repair and Reconstruction, Division of Plastic, Aesthetic and Reconstructive Surgery, Department of Surgery, Medical University of Graz, 8036 Graz, Austria

**Keywords:** hypertrophic scar, fibrosis, animal model, inflammation, hypoxia, Duroc pig, porcine scar, burn scar, TGF-b

## Abstract

Hypertrophic scars continue to be a major burden, especially after burns. Persistent inflammation during wound healing appears to be the precipitating aspect in pathologic scarring. The lack of a standardized model hinders research from fully elucidating pathophysiology and therapy, as most therapeutic approaches have sparse evidence. The goal of this project was to investigate the mechanisms of scar formation after prolonged wound inflammation and to introduce a method for generating standardized hypertrophic scars by inducing prolonged inflammation. Four wound types were created in Duroc pigs: full-thickness wounds, burn wounds, and both of them with induced hyperinflammation by resiquimod. Clinical assessment (Vancouver Scar Scale), tissue oxygenation by hyperspectral imaging, histologic assessment, and gene expression analysis were performed at various time points during the following five months. Native burn wounds as well as resiquimod-induced full-thickness and burn wounds resulted in more hypertrophic scars than full-thickness wounds. The scar scale showed significantly higher scores in burn- and resiquimod-induced wounds compared with full-thickness wounds as of day 77. These three wound types also showed relative hypoxia compared with uninduced full-thickness wounds in hyperspectral imaging and increased expression of *HIF1a* levels. The highest number of inflammatory cells was detected in resiquimod-induced full-thickness wounds with histologic features of hypertrophic scars in burn and resiquimod-induced wounds. Gene expression analysis revealed increased inflammation with only moderately altered fibrosis markers. We successfully created hypertrophic scars in the Duroc pig by using different wound etiologies. Inflammation caused by burns or resiquimod induction led to scars similar to human hypertrophic scars. This model may allow for the further investigation of the exact mechanisms of pathological scars, the role of hypoxia and inflammation, and the testing of therapeutic approaches.

## 1. Introduction

Scars are the body’s physiological consequence of deep dermal injury. Ideally, they are flat and barely visible. However, under certain circumstances, excessive scarring can occur, and hypertrophic scars develop. One reason for the formation of hypertrophic scars is an overproduction of collagen/connective tissue fibers mediated by increased expression of profibrotic factors such as transforming-growth-factor beta (*TGFb*) 1 and 2 [1]. The hypertrophic scar tends to bulge and often appears red and raised significantly above the surrounding skin level. Symptoms such as itching and pain, as well as joint contractures caused by the scars that impair movement can significantly affect patients’ quality of life [2]. Few of the various treatments available are based on actual scientific evidence, not least because of the lack of a standardized model for hypertrophic scars that provides deeper insight into the pathophysiology [3].

Although the pathophysiology of hypertrophic scars has not been fully elucidated, several factors appear to play an important role. These factors include the type and depth of injury [4]; after burns, these pathologic scars occur with a frequency of up to 70%, whereas the frequency after surgical procedures is about 30% [5]. Therefore, a possible reason can be found in the persistent and increased inflammatory response [6,7,8]. Inflammation is an integral part of wound healing, as it is essential for physiologic wound debridement and adequate pathogen defense mechanisms. While wound healing is generally self-regulating, dysregulations in the wound healing cascade characterized by prolonged and/or increased inflammatory responses can lead to increased tissue fibrosis and an imbalance between immature collagen III and mature collagen I [9,10]. *TGFb* has been identified as an important player in several wound healing phases and the regulation of tissue fibrosis. It is responsible for the attraction of neutrophils, the resolution of inflammation, cell differentiation, proliferation and migration, and the balance of collagen deposition [11]. Recent studies investigated the role of *TGFb*, hypoxia and hypoxia-inducible factor 1a (*HIF1a*) in inflammation and found a correlation between increased hypoxia as well as increased tissue fibrosis [12,13,14,15]. These findings may be a possible explanation for the higher prevalence of pathologic scarring in burns compared with surgical trauma mentioned above, as thermal trauma induces much higher systemic and local inflammation and consecutive hypoxia [16,17,18,19]. However, many details of the pathophysiology have not been fully elucidated, and to date, persistent inflammation is considered the most important factor responsible for pathologic scarring.

Our group recently induced prolonged inflammation in a porcine model to study delayed wound healing using the immunomodulatory drug resiquimod, which acts as an agonist on Toll-like receptor (TLR) 7/8 [20]. This prolonged inflammation resulted in delayed wound healing, which is considered to be a crucial factor in pathological scar formation [8,20]. In the present study, we transferred the findings from this project in order to develop a new model for the formation of standardized hypertrophic scars.

To date, there is no representative and properly established model that allows a consistent investigation of the exact mechanisms and a thorough and standardized testing of treatment modalities. In this study, the Duroc pig was chosen as a more suitable model for further research on excessive scarring because of the similarity of its scars to human hypertrophic scars [21,22].

This project will help to elucidate the pathophysiology of hypertrophic scars, to investigate what happens beyond inflammation, and to establish a way to create standardized hypertrophic scars by using resiquimod.

## 2. Results

### 2.1. Resiquimod-Induced Wounds Develop Hypertrophic Scars

Forty-four wounds were applied to the six Duroc pigs, resulting in a total of eleven wounds per type (burn wound (BW), full thickness wound (FT), burn wound with resiquimod (BWR), full thickness wound with resiquimod (FTR)). All wounds were fully epithelialized after four weeks, although the wounds induced with resiquimod (BWR, FTR) showed a higher inflammatory response during healing. The induced wounds showed a lower amount of granulation tissue, increased necrosis and delayed re-epithelialization, and inflamed wound edges. The non-induced wounds showed no relevant signs of inflammation in the full-thickness wounds and moderate inflammation in the burn wounds.

**Figure 1:**
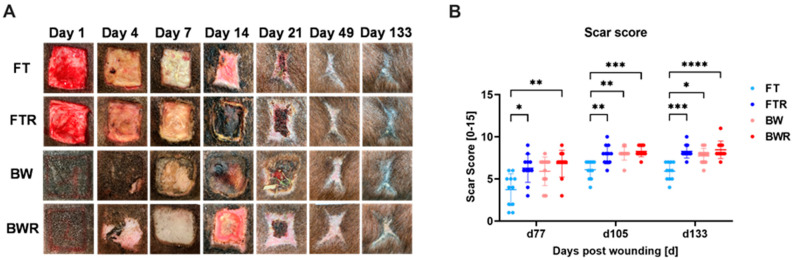
Burn injury and resiquimod-induced inflammation in full-thickness wounds lead to hypertrophic scarring. (**A**) Representative images of wound appearance during the study course. Wounds were regularly examined and photographed. A total of 44 wounds were created with eleven wounds per type. (**B**) The scar score of all wounds based on the modified Vancouver Scar Scale showed that resiquimod-induced full-thickness wounds and burn wounds had higher scores as early as day 77, whereas differences between non-induced wounds were not apparent until day 105. Resiquimod-induced wounds had higher scar scores than non-induced wounds throughout the study course. Data are presented as mean ± SD. Statistical significance was determined using the Kruskal-Wallis test and a Tukey post hoc test. * = *p* < 0.05, ** = *p* < 0.01, *** = *p* < 0.001, **** = *p* < 0.0001, FT = Full-thickness wound, FTR = Full-thickness wound with resiquimod, BW = Burn wound, BWR = Burn wound with resiquimod.

Immediately after wound closure, pronounced scars began to form, with signs of hypertrophy visible on day 77. Scar assessment with a modified Vancouver Scar Scale was performed on days 77, 105, and 133. Significantly higher scores were achieved by induced wounds (BWR vs. FT: *p* = 0.0012; FTR vs. FT: *p* = 0.036) as early as day 77. Further differences between uninduced wounds became apparent at day 105, with BW achieving higher scores than FT (BW vs. FT: *p* = 0.0019; BWR vs. FT: *p* = 0.0002; FTR vs. FT: *p* = 0.0080). These differences persisted until the end of the study at day 133 (BW vs. FT: *p* = 0.0150; BWR vs. FT: *p* < 0.0001; FTR vs. FT: *p* = 0.0002). Given the small sample size, the effect size (Cohen’s d) was additionally calculated for BWR vs. BW; the resulting effect size of 0.749 is considered a very large effect [23,24]. Figure 1 shows the exemplary healing process for each wound type and the corresponding scar score.

### 2.2. Wound Oxygenation Is Impaired after Burn Injury and Resiquimod-Induced Wounds

**Figure 2:**
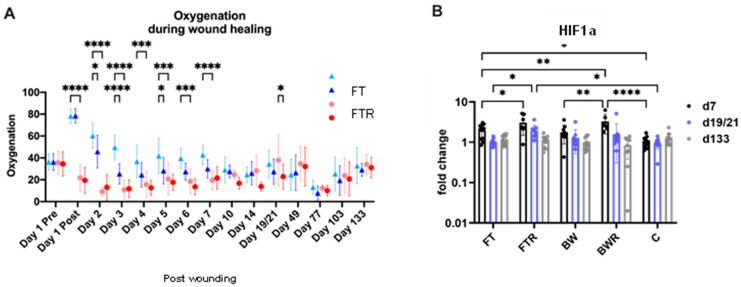
Burn injury and resiquimod-induced inflammation in full-thickness wounds result in relative hypoxia. (**A**) Oxygenation levels in different wound types examined by hyperspectral imaging showed hyperoxygenation of full-thickness wounds immediately after wounding with resiquimod-induced full-thickness wounds rapidly reaching the relative hypoxia levels of burn wounds. Oxygenation levels converged 10 days after wounding. (**B**) Analysis of HIF1a expression levels in wound biopsies showed higher expression in resiquimod-induced wounds than in non-induced full thickness wounds at day 7, and the difference between induced and non-induced wounds persisted until day 21. Data are presented as mean ± SD. Statistical significance was determined using the two-way-ANOVA test and a Tukey post hoc test. * = *p* < 0.05, ** = *p* < 0.01, *** = *p* < 0.001, **** = *p* < 0.0001, FT = Full-thickness wound, FTR = Full-thickness wound with resiquimod, BW = Burn wound, BWR = Burn wound with resiquimod, C = Control biopsies.

Hyperspectral imaging allows for the assessment of tissue oxygenation below the wound surface. Immediate relative hypoxia was observed in burn wounds compared with reactive hyperoxygenation in full-thickness wounds. Application of resiquimod to full-thickness wounds resulted in rapid elimination of hyperoxygenation at day 4, approximating hypoxia levels of burn wounds (FTR vs. BW/BWR: *p* > 0.05), with differences between induced and non-induced wounds being significant as early as day 2 (*p* = 0.021). Normalization of oxygenation in non-induced full-thickness wounds was achieved on day 10, and no significant differences were seen thereafter. Similarly, HIF1a expression showed marked upregulation in resiquimod-induced wounds (BWR, FTR), whereas FT showed only slight upregulation compared with control biopsies (C). The higher HIF1a expression remained significant in FTR wounds compared with FT wounds until day 19/21 after wounding, whereas expression levels in the other wound types had normalized by that time. Figure 2 shows the course of wound oxygenation and HIF1a expression levels. 

### 2.3. Resiquimod-Induced Wounds Produced Scars with Histologic Features of Human Hypertrophic Scars

**Figure 3:**
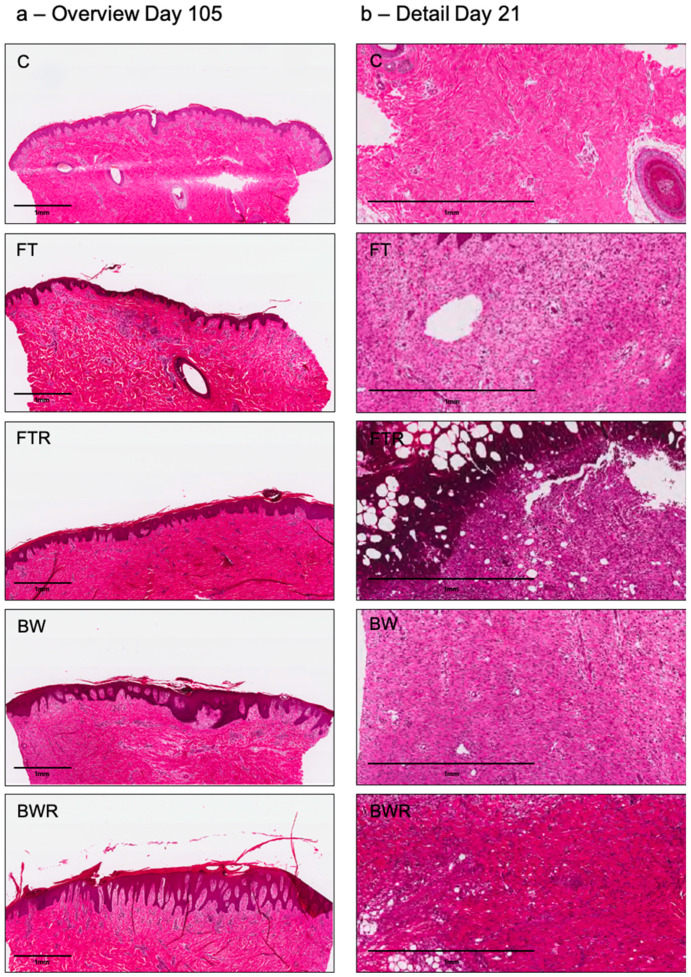
Representative histological sections of the different wound types in hematoxylin/eosin staining (Top to bottom: Control (C), full-thickness wound (FT), full-thickness wound with resiquimod (FTR), burn wound (BW), burn wound with resiquimod (BWR)). The left column (**a**) shows an overview of the biopsies at day 105 with significantly thicker epidermis in the burn wound and the resiquimod-induced wounds typical of human hypertrophic scars. The right column (**b**) shows a larger dermal section of the respective wound types on day 21. Upon quantification (not shown, see Figure 4), inflammatory cell invasion was significantly higher in FTR wounds compared to all other wound types.

Histologic analysis revealed a thicker dermis and epidermis, typical of hypertrophic scars, in scars of burn wounds and wounds induced by resiquimod. The latter showed epidermal ridges reaching deep into the dermal layers as compared to scars of non-induced wounds. In FT wounds, the collagen appeared rather wavy and organized, whereas in hyperinflammatory wounds (FTR, BW, BWR), the collagen appeared disorganized with the occurrence of collagen nodules. The left column of Figure 3a shows exemplary histological sections of the different scars.

### 2.4. Burn Injury and Resiquimod Induction Promoted Inflammation

**Figure 4:**
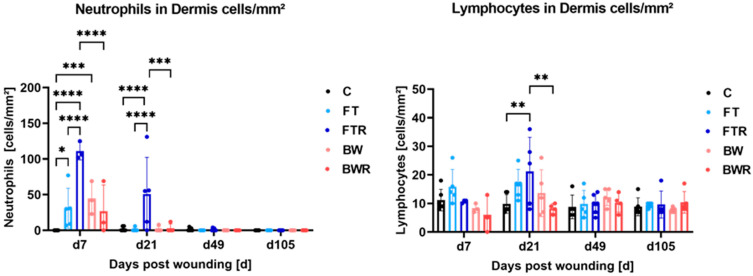
Resiquimod-induced inflammation and burn injury increased infiltration of neutrophils and lymphocytes into the wound. Immune cells were counted in hematoxylin/eosin-stained sections of biopsies on days 7, 21, 49, and 105. Neutrophils and lymphocytes were significantly increased in full-thickness resiquimod-induced wounds. While cell counts were increased in the other wound types compared with control biopsies, they showed no differences among themselves. Data are presented as mean ± SD. Statistical significance was determined using two-way ANOVA and a Tukey post hoc test. * *p* < 0.05, ** *p* < 0.01, *** *p* < 0.001, **** = *p* < 0.0001, C = Control, FT = Full-thickness, FTR = Full-thickness with resiquimod, BW = Burn wound, BWR = Burn wound with resiquimod.

Histological analysis revealed significantly higher numbers of neutrophils in the dermis on day 7 and day 21, particularly in FTR wounds. On day 7, FT, FTR and BWR wounds showed significantly more neutrophil infiltration than the control group (*p* = 0.0127, <0.0001 and 0.0010, respectively). The FTR group also had significantly more neutrophils than the FT, BW and BWR groups (*p* for all <0.0001). At day 21, the FTR group had significantly more neutrophils in the dermis than the control, FT, BW, and BWR groups (*p* < 0.0001 for C, FT, and BW, and *p* = 0.0002 for BWR). There were no significant differences in wound types other than FTR. Differences in the mean number of lymphocytes in the dermis of FTR wounds (21.2 cells/mm^2^) and in BWR wounds (8.25 cells/mm^2^; *p* = 0.0016) and in control biopsies (9.83 cells/mm^2^; *p* = 0.0023) were noted at day 21. All other results regarding the number of lymphocytes did not show statistical significance. Figure 3b (right column) shows exemplary dermal sections of the different wound types on day 21. Figure 4 depicts the distribution of inflammatory cells.

**Figure 5:**
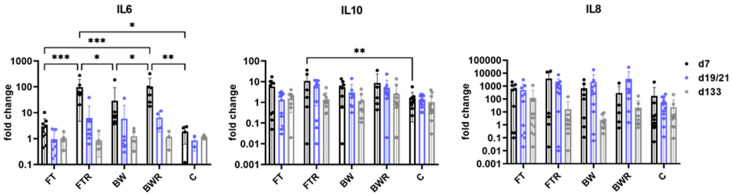
Burn injury and resiquimod induction moderately increased the expression of IL6 and IL10 but had no effect on the expression of IL8. The relative mRNA expression levels of IL6, IL8, and IL10 were determined by qPCR, with target gene expression normalized to the averaged expression of the housekeeping gene YWHAZ. Data are presented as mean (bars) + standard deviation (whiskers). Statistical significance was determined using the two-way ANOVA and corrected for multiple comparisons using Tukey’s method. * *p* < 0.05, ** *p* < 0.01, *** *p* < 0.001, C = Control, FT = Full-thickness, FTR = Full-thickness with resiquimod, BW = Burn wound, BWR = Burn wound with resiquimod.

In the gene expression analysis (see Figure 5), the pro-inflammatory mediator IL6 showed significantly higher expression in resiquimod-induced wounds (FTR, BWR) than in placebo-treated BW (*p* = 0.039 and 0.027, respectively) and in placebo-treated FT wounds (*p* = 0.001 and *p* = 0.001, respectively). No significant difference between induced wound types was seen (*p* > 0.05). These differences were evident only in the initial phase of the study (day 7) and leveled off over time. The non-induced burn wounds showed no significantly different expression than the controls (*p* > 0.05 for both). The anti-inflammatory mediator IL10 showed a slightly higher expression in FTR than in the control on day 7 (*p* = 0.006). No other differences were observed in the IL10, and none at all in the IL8 analysis.

### 2.5. Wound Remodeling Was Moderately Affected by Burn Injury or Resiquimod Induction

**Figure 6:**
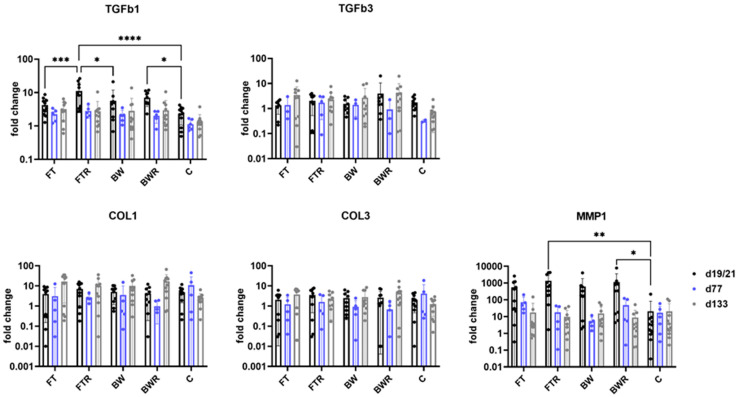
Resiquimod induction increased the expression of TGFb1 and MMP1. The highest expression was observed in FTR wounds. The other remodeling factors TGFb3, COL1 and COL3 showed no significant differences at the time points examined. The relative mRNA expression levels of TGFb1, TGFb3, COL1, COL3 and MMP1 were determined by qPCR, with target gene expression normalized to the averaged expression of the housekeeping gene YWHAZ. Data are presented as mean (bars) + standard deviation (whiskers). Statistical significance was determined using the two-way ANOVA and corrected for multiple comparisons using Tukey’s method. * *p* < 0.05, ** *p* < 0.01, *** *p* < 0.001, **** *p* < 0.0001, C = Control, FT = Full-thickness, FTR = Full-thickness with resiquimod, BW = Burn wound, BWR = Burn wound with resiquimod.

Gene expression analysis revealed a significant increase only for TGFb1 in resiquimod-induced wound types compared with controls. Resiquimod-induced full-thickness wounds also showed significant overexpression compared with non-induced full-thickness wounds and burn wounds on day 19/21. Matrix metallopeptidase 1 (MMP1) also showed significantly increased expression in resiquimod-induced wounds compared with controls. TGFb3, collagen 1 (COL1), and 3 (COL3) did not show significantly different expression patterns at all the time points examined. Figure 6 shows the results of gene expression analysis of remodeling factors.

## 3. Discussion

The whens and hows of the occurrence of hypertrophic scars are not yet fully understood. Most researchers agree that persistent inflammation is an important factor [4,9,25,26,27], but the exact mechanisms are still unclear, and there is little evidence for most therapeutic approaches [3]. One reason for this may be the lack of a satisfactory standardized model [2,28,29]. In this project, we demonstrated that the induction of prolonged inflammation in a standardized manner results in scars resembling human hypertrophic scars. Moreover, a new model for hypertrophic scars in the Duroc pig was developed. Resiquimod-induced full-thickness wounds (FTR) showed the most prominent scars as well as the most pronounced histological appearance and altered gene expression. Furthermore, burns, which may be considered the preferred wound type for the development of hypertrophic scars, also resulted in pronounced hypertrophic scars, without significant differences from induced full-thickness wounds. Full-thickness wounds showed scarring not as pronounced as hyperinflammatory wounds. We were thus able to confirm that burns trigger pathologic scarring. This effect can even be increased by induction of wounds with resiquimod. These insights may allow for the avoidance of burns in future animal studies for hypertrophic scars, which would be a valuable refinement in terms of the 3Rs (Replacement, Reduction, Refinement) [30].

Developing this model, we were able to produce scars that had macroscopic similarities to human hypertrophic scars with dense, fibrotic, and contracted aspects. In previous studies, our study group used the immunomodulatory drug resiquimod to induce persistent inflammation and simulate a non-healing wound [20]. In the current setup, the drug was used on full-thickness wounds and burn wounds to examine the local differences in resulting scars in the Duroc pig. While initial studies by Silverstein et al. [31] and others using the red Duroc pig used dermatome wounds and reportedly produced hypertrophic scars (although difficult to reproduce) [2,22,32,33], a more recent study followed the trend of occurrence of hypertrophic scars in humans and successfully produced more prominent scars in burn wounds [21]. Burn wounds are an important wound etiology in scar formation, as the wound conditions following burns trigger hypertrophic scarring by themselves already. In our study, we were able to show this and further amplify these effects in the Duroc pig. Most previous studies used female Duroc pigs, whereas we used castrated male Duroc pigs. This decision was made for practical reasons, as there were not enough female Duroc pigs available from breeders at the time the experiment was planned. In addition, confounding effects from hormonal changes due to the menstrual cycle could be avoided, although Gallant et al. reported no relevant difference in wound healing and scarring parameters between female and castrated male Duroc pigs [34].

In our study, the degree of hypertrophy was primarily assessed using a modified version of the Vancouver Scar Scale, which is one of the most commonly used rating scales for assessing scars [35]. The fact that the observers evaluating the scars were blinded to the wound types allowed for a bias-free and valid assessment. As expected, scar scores for BW were higher than those for FT wounds, i.e., “more hypertrophic”. Interestingly, the scores obtained from resiquimod wounds (FTR, BWR) were as high as those obtained from “normal” burn wounds (BW), leading to the conclusion that resiquimod-induced prolonged inflammation is a valid method to induce hypertrophic scars. The difference between burn wounds and burn wounds induced by resiquimod was not as distinct as the difference between induced and non-induced full-thickness wounds, especially when it comes to macroscopic assessment and oxygenation levels. This supports the common theory that a burn with its associated features already triggers pathological scarring (and the corresponding signaling pathways) on its own [36,37,38], whereas hyperinflammation induced by resiquimod mimics, in a sense, the effects of a burn on wounds. Given the already high inflammatory (and hypoxia) response in an uninduced burn, the resulting effects do not appear to be as pronounced as in full-thickness wounds, which, when uninduced, undergo “normal” wound healing. *HIF1a* expression in induced burn wounds was significantly higher than in non-induced burn wounds, but scar scores at day 133 did not show significantly higher scores for the induced burn wounds than for the non-induced wounds. Upon closer inspection, a trend towards higher values can be seen, resulting in a large effect size of 0.749 (Cohen’s *d*) and highlighting the role of hypoxia-induced inflammation in pathologic scar formation. These results also again show that burns themselves without further induced inflammation already trigger pathologic scar formation.

Moreover, the scores of FTR and BWR were already significantly higher than those of FT on day 77, whereas the difference between FT and BWR was not evident until day 105. Thus, resiquimod enhances the signs of hypertrophic scars at an earlier time point than the burn-induced effects. The timing of scar discrimination is similar to other studies (approximately three months after wounding) [21,33,39], although the scars in our study were significantly different slightly earlier. Another study by Gallant et al. reported a “raised, dense, fibrotic appearance” as early as between days 42 and 56, which had improved by day 70 again [34]. However, these differences in timing should be interpreted with caution, as the timing of the assessment may have been chosen for logistical reasons without considering each day between assessments. In addition, the wound model used by Gallent et al. compared deep dermal wounds with full-thickness wounds. Another possible reason could be the genetic variance of Duroc pigs due to different breeders and backgrounds.

Hyperspectral imaging allows the assessment of tissue perfusion and oxygen saturation in tissues of a few millimeters’ depth by the remission of light of different wavelengths. Using this technique, we were able to demonstrate, on the one hand, an immediate decrease in oxygen saturation in burn wounds, which is a well-known consequence of burn injuries [40]. No difference was detected between BW and BWR wounds. On the other hand, the immediate increase in tissue oxygen saturation after wounding in full-thickness wounds (FT and FTR) indicated increased blood flow and blood pooling. However, upon resiquimod induction, the level of tissue oxygenation rapidly approached that of the burn wounds, whereas oxygenation in the FT wounds did not reach its baseline level for ten days. Resiquimod is a modulator that acts as an agonist of TLR7/8. TLRs play an essential role in wound healing and an increase in TLR can lead to a prolongation of wound healing time [20,41,42,43]. Resiquimod thus mimics the effects of burns, with prolonged healing time (as in burns) considered a driving factor for pathological scarring. The present study suggests that the agonistic function of TLR7/8 also induces hypoxia or at least suppresses reactive hyperperfusion of the full-thickness wound leading to relative hypoxia. The role of hypoxia in myofibroblast differentiation and scar formation has been discussed by several authors [44,45,46,47], and the link between hypoxia and inflammation is also well established [48,49,50]. Our findings of relative hypoxia together with the increased scar score levels in the underperfused wounds suggest that hypoxia is the precipitating condition leading to increased and sustained inflammation, which ultimately results in hypertrophic scarring.

Our histologic analysis revealed typical aspects of hypertrophic scars, particularly in BWR, FTR, and also BW wounds, with thickened epidermis and the occurrence of disorganized collagen and collagen nodules [2,34,51]. The presence of lymphocytes and neutrophils in the dermis is further evidence of increased inflammation. The concentration is highest in FTR wounds, highlighting the neutrophil activation and priming function of the TLR7/8 agonist resiquimod [52,53].

Consistent with histologic quantification, gene expression analysis revealed that resiquimod-induced sustained inflammation resulted in upregulation of inflammatory factors with only a moderate effect on remodeling factors. The inflammatory markers *IL6* and *IL10* showed significant upregulation, especially in FTR wounds, lower also in BWR and BW wounds. This upregulation is mediated by resiquimod, for which exactly this effect has been demonstrated [54,55]. This increased and prolonged relative hyperinflammation cause by a burn or in this case resiquimod induction demonstrates the “burn-mimicking” effect of resiquimod. Transferred to a clinical setting, this hyperinflammation that was still visible seven days after the wound infliction, emphasizes the need for early surgery to remove the inflammation-triggering tissue. Early excision has been shown to significantly reduce scarring and increase survival in burn patients [56,57,58]. Alternatively, if surgery is not indicated or possible, burn wounds should not be left to themselves, but consequent conservative care with specifically designated burn wound dressings is required to at least slow down the excessive hyperinflammation [25,59].

Interestingly, the hypoxia-associated factor *HIF1a* showed a more pronounced response than the inflammatory markers at the investigated timepoints, supporting the above-mentioned hypothesis that hypoxia is the driving factor in this scar model and inflammation is a relevant but possibly subordinate aspect. The occurrence of oxidative stress as a result of wound hypoxia in BW, FTR, and BWR wounds also contributes to the enhancement of further inflammation, as the TLR8-mediated response is amplified by oxidative stress [60,61,62,63]. An interesting approach to be investigated in further studies would be the influence on scarring and inflammatory as well as hypoxia parameters by the induction of another inflammatory boost after full re-epithelialization. This could be performed by using lipopolysaccharides that were used in prior studies to exacerbate inflammation [64]. *MMP1*, as a fibrotic regulator, showed only minor changes in the form of slight upregulation that quickly returned to normal levels, as shown by previous studies [34,39]. Our model did show increased expression of *TGFb1* in resiquimod-treated wounds but did not confirm significantly increased levels of the remodeling factors *TGFb3*, *COL1*, and *COL3*, as would have been expected based on macroscopic fibrosis. FTR and lower also BWR wounds were the only wounds that showed significantly increased *TGFb1* expression compared with control wounds. These wounds were the most responsive in terms of inflammatory response on both gene expression and histologic analysis. Concluding, this project allowed further insight in the circumstances that lead to hypertrophic scar formation; we could show that burn wounds cause more hypertrophic scars than full-thickness wounds, and induction by resiquimod mimics or even amplifies these effects.

### Limitations

This study had some inherent limitations. First, we performed two sets of experiments with three Duroc pigs each. Due to the COVID-19 pandemic, the breeder of the first series went out of the market so the Durocs in the second series came from a different breeder. We observed slight differences in macroscopic appearance of scars as well as in histological and gene expression analysis between the two series, although all six Duroc pigs were declared as purebred. Genetic variance could therefore be a relevant confounding factor in this project. Another limitation was the timing of testing and examinations. Since these were limited by the long duration of the experiment, important information/developments might have been missed that would have been visible at other timepoints. Obviously, given the dimension of the experiments, other relevant parameters that could provide interesting aspects were not investigated in this project.

## 4. Materials and Methods

All animal work was reviewed and approved by the responsible authority, the Federal Ministry Republic of Austria, Education, Science and Research (BMBWF-66.010/0116-V/3b/2019).

### 4.1. Animal Model

Six castrated male red Duroc pigs (age at study entry: 8–12 weeks, weight: 15.83 kg ± 5.14 kg) were used in this study to avoid confounding effects of hormonal fluctuations on inflammation and scarring. Prior to any study-related activity, the animals were acclimatized to the research facility for two weeks and had access to food and water ad libitum for the duration of the experiment. All animal housing and handling was carried out in a species-appropriate manner with veterinarians and/or veterinary qualified personnel present at all times. The backs of the pigs were shaved one day before wounding. On the day of wounding, the pigs were anaesthetized for the duration of the wounding using 0.5 mg/kg midazolam (Midazolam Accord, Accord Healthcare Limited, Devon, UK), 10 mg/kg ketamine (Ketasol, Graeub, Bern care Limited, Devon, UK), 0.2 mg/kg butorphanol (Butomidor, Richter Pharma AG, Vienna, Austria) and 2 mg/kg azaperone (Stresnil, Elanco GmbH, Bad Homburg vor der Höhe, Germany). After surgery, analgesia was administered by fentanyl patches of 25 to 50 µg/h (depending on individual need). Each pig received 6 full-thickness wounds (3 × 3 cm) on the back. The animals were wounded by excision and by full-thickness burns. Five of the six pigs received four wounds per type (total of eight wounds) and one pig received two wounds per type (total of four wounds) because of its smaller size and weight. Half of the wounds of each wound type were treated with 1 g of 0.045% resiquimod ointment (R-848, Invivogen, San Diego, CA, USA), an immunomodulatory, inflammation-inducing drug previously used by our study group to produce a sustained inflammatory response in porcine wounds [20]. The exact composition of the ointment is described below. Treatment with the ointment was performed for the following six consecutive days, while the other half was treated with 1 g of a placebo ointment without active ingredient. Wounds were 3 × 3 cm in size and were located on the bilateral flanks with varying location of the wound types on the pigs to avoid confounding effects due to the location of the wounds. Excisional wounds were created with a scalpel, and burn wounds were created by contact with a 200 °C stainless steel cuboid for 30 s under general anesthesia. Reapplication of the ointment was performed daily for the first 6 days. Dressing changes were performed every other day thereafter until day 14, when no further dressing changes were required. Biopsy punches and imaging of the wounds/scars were performed as described below. The study was concluded after day 133 (5 months).

#### 4.1.1. Preparation of the Topical Formulations

Resiquimod was formulated into a cream following US 2007/0264317 A1. Briefly, 0.45 mg resiquimod was dissolved in endotoxin-free water under vortexing for the preparation of 1g ointment. The aqueous phase further comprised 0.5% (*w*/*w*) xanthan gum (Lactan, Graz, Austria) and 3.4% (*w*/*w*) polysorbate 60 (Sigma Aldrich, Munich, Austria). Resiquimod was substituted with water in equal parts for the placebo ointment. The lipophilic phase, consisting of 8.85% (*w*/*w*) oleic acid (Merck, Darmstadt, Germany), 3.6% (*w*/*w*) stearic acid, 2.63% (*w*/*w*) cetyl alcohol, 3.71% (*w*/*w*) stearyl alcohol, 3.59% (*w*/*w*) Vaseline white, 0.72% (*w*/*w*) sorbitan monostearate, and 2.39% (*w*/*w*) glycerol was heated to 70 °C. Both phases were mixed together at the same temperature, stirred cold, and the preservative consisting of 0.2% (*w*/*w*) methylparaben, 0.02% (*w*/*w*) propylparaben, and 2% (*w*/*w*) benzyl alcohol was added. Unless otherwise stated, the materials were purchased from Herba Chemosan, Graz, Austria.

#### 4.1.2. Scar Scoring

After complete wound closure, scars were assessed on days 77, 105, and 133 using a modified version of the Vancouver Scar Scale (VSS). Pigmentation (0–2 points), vascularity (0–3 points), pliability (0–4 points), height (0–3 points), and size (0–3 points) were scored by two independent examiners without their knowledge of which scar belonged to which wound type (blinded). The sum of the parameters resulted in a value between 0 and 15 for each scar. Earlier assessments were not performed because the scars did not show any noticeable changes before day 77.

### 4.2. Imaging

Regular photographic imaging and hyperspectral imaging were performed on days 0–7, 10, 14, 19/21, 49, 77, 105. TIVITA^®^ Wound (500–1000 nm, Diaspective Vision GmbH, Am Salzhaff-Pepelow, Germany) was used for hyperspectral imaging. Hyperspectral imaging allows the assessment of tissue perfusion, oxygen saturation and hemoglobin content by the remission of light of different wavelengths of the illuminated tissue. The results are visualized by color-coded images on a computer, translating each parameter into a standardized numerical value that allows the quantification of the parameter. The here indicated parameter is the NIR (Near-Infrared Perfusion Index), a dimensionless value allowing relative tissue perfusion assessment in up to 4–6 mm depth.

### 4.3. Punch Biopsies

Eight-millimeter punch biopsies of each wound/scar were taken under analgosedation on days 0 (skin excision only), 7, 19/21, 49, 77, 105, and 133. In addition, two punch biopsies were taken from the cranial and caudal back of the animals at each of the above time points to serve as negative controls. The biopsies were divided and half of them were stored in formalin for histological processing, whereas the other half was frozen in liquid nitrogen for further gene expression analysis. After the procedure, pain management with fentanyl patches of 25 to 50 µg/h (depending on individual needs) was administered.

### 4.4. Histology

The histological workup was conducted as described in [20]. Biopsies were fixed in 10% formalin solution before being embedded in paraffin. They were cut into slices three micrometers thick and stained with hematoxylin and eosin.

All stained tissue sections were examined for the presence of neutrophils and lymphocytes in the dermal scar region. Cell counting was performed via microscope with an ocular field diameter of 0.5 mm at 400× magnification (Nikon MICROPHOT-FXA, Nikon, Tokyo, Japan). Each ocular field corresponded to a high-power field. For each case, four randomly selected high-power fields within the dermis were evaluated. The counts of each cell type were then summed, resulting in the respective cell counts/mm^2^. Counting and data collection were performed by a trained pathologist who was blinded to the different scar etiologies. Sections that contained air pockets were excluded from analysis.

### 4.5. Gene Expression Analysis

Porcine skin biopsies were homogenized in QIAzol using innuSPEED Lysis tubes and the Speedmill Plus instrument (Analytik Jena GmbH, Jena, Germany). RNA from the homogenized tissue was isolated using the RNeasy Mini Kit (Qiagen, Hilden, Germany) according to the manufacturer’s protocol. NanoDrop One microvolume spectrophotometer (Thermo Fisher Scientific, Waltham, MA, USA) was used to quantify RNA. For gene expression analysis, 1 µg of total RNA was reverse transcribed into single-stranded cDNA using the iScript™ Reverse Transcription Supermix (Bio-Rad Laboratories, Inc., Hercules, CA, USA). Commercially available PrimePCR™ probe assays for the genes of interest and the housekeeping gene (Table 1) were purchased from Bio-Rad and the TaqMan™ Gene Expression Master Mix from Thermo Fisher Scientific. Real-time PCR was performed on a Bio-Rad CFX384 cycler. Relative target gene expression was normalized to the reference gene YWHAZ and calculated using the ΔΔCq method. All samples were calibrated with a blank skin sample. N-fold expression levels are shown as mean (bar) and standard deviation (whiskers). Cq values of duplicates deviating more than one cycle were excluded from further analysis.

#### Statistical Analysis

Statistical analysis was performed using Prism 9 (GraphPad Software, San Diego, CA, USA). Data were analyzed using mean, median, standard deviation, and other descriptive statistics variables. To describe inferential statistics, ANOVA, Kruskal–Wallis test and Tukey’s post hoc test were used. Significance was set at *p* < 0.05. In addition, Cohen’s *d* was calculated to evaluate the effect size.

## 5. Conclusions

In this study, we investigated mechanisms of hypertrophic scar formation after prolonged inflammation and introduced a novel model for standardized hypertrophic scars in the Duroc pig. We studied four different wound types, of which the full-thickness wounds in which inflammation was prolonged by the addition of resiquimod showed the most promising results. The resulting scars showed similarities to human hypertrophic scars. They were dense, fibrotic, and contracted, showed histologic thickening of the epidermis and dermis infiltrated with inflammatory cells, and exhibited disorganized collagen that occurred in nodules. The addition of resiquimod to the full-thickness wound resulted in a rapid decrease in tissue oxygenation, as seen in hyperspectral imaging, which closely resembles tissue oxygenation in burn wounds. Gene expression analysis revealed increased expression of *HIF1a*, leading to the suggestion that hypoxia is the driving factor in promoting inflammation and thus the appearance of hypertrophic scars. This model allows for the generation of solid hypertrophic scars without burns, which allows for a more standardized, possibly more humane and thus refined animal experiment in terms of the 3Rs. Future studies should further refine this model potentially by administering a second inflammatory burst and by exploring the precise role of hypoxia in the pathophysiology of hypertrophic scars and as a potential therapeutic target.

## Figures and Tables

**Table 1:** PrimePCR™ Probe Assays (Bio-Rad Laboratories, Inc., Hercules, CA, USA).

Gene	Name; Commonly Used Aliases	Cat. No.
*COL1A1*	collagen type I alpha 1 chain	qSscCEP0041702
*COL3A1*	collagen type III alpha 1 chain	qSscCIP0024827
*HIF1a*	hypoxia inducible factor 1 subunit alpha	qSscCIP0025745
*IL6*	interleukin 6	qSscCEP0035848
*IL8*	interleukin 8	qSscCEP0032327
*IL10*	Interleukin 10	qSscCEP0028429
*MMP1*	matrix metallopeptidase 1	qSscCEP0032832
*TGFb1*	transforming growth factor beta 1	qSscCIP0039450
*TGFb3*	transforming growth factor beta 3	qSscCIP0040113
*YWHAZ*	tyrosine 3-monooxygenase/tryptophan 5-monooxygenase activation protein zeta	qSscCIP0027700

## Data Availability

The datasets generated and analyzed during the current study are available from the corresponding author on reasonable request. All data generated and analyzed during this study are included in this published article.
